# Rottlerin as a novel chemotherapy agent for adrenocortical carcinoma

**DOI:** 10.18632/oncotarget.15221

**Published:** 2017-02-09

**Authors:** Yi Zhu, Minjie Wang, Xu Zhao, Lei Zhang, Yigao Wu, Bangqi Wang, Weilie Hu

**Affiliations:** ^1^ Third Military Medical University, Chongqing, P.R. China; ^2^ Guangzhou General Hospital of Guangzhou Military Command, Guangzhou, Guangdong, P.R. China; ^3^ No. 422 Hospital of PLA, Zhanjiang, Guangdong, P.R. China; ^4^ Southern Medical University, Guangzhou, Guangdong, P.R. China

**Keywords:** adrenocortical carcinoma, rottlerin, Wnt/β-catenin, anticancer agent

## Abstract

Adrenocortical carcinoma (ACC) is a rare, but aggressive endocrine malignancy with a generally poor clinical outcome. There is no effective therapy for advanced and metastatic ACC. In our study, we found that an existing drug (rottlerin) exerted its tumour-suppressive function in ACC. Specifically, rottlerin inhibited cellular proliferation of ACC cell lines (NCI-H295R and SW-13) in a dose- and time-dependent manner. We also found that rottlerin induced cell apoptosis and promoted G0/G1 cell cycle arrest in ACC cell lines. The cellular migration and invasion of ACC cell lines were decreased after treatment with rottlerin. Further, the molecular expression of lipoprotein receptor related protein 6 (LRP6) and β-catenin were down-regulated in rottlerin-treated ACC cells, which indicated that Wnt/β-catenin signaling was involved in the tumour-suppressive function of rottlerin. To further confirm the anti-tumour function of rottlerin, a nude mouse ACC xenograft model was used. The xenograft growth curves and TUNEL assays demonstrated that rottlerin inhibited proliferation and induced apoptosis in the ACC xenograft model. Furthermore, we verified that rottlerin down-regulated the expression of LRP6 and β-catenin *in vivo*. The ACC cell line and xenograft mouse model data indicated that rottlerin significantly inhibited proliferation and induced apoptosis of ACC cells, likely via suppression of the Wnt/β-catenin signaling pathway. Our study indicated the potential therapeutic utility of rottlerin as a novel and potential chemotherapeutic agent for ACC.

## INTRODUCTION

Adrenocortical carcinoma (ACC) is a rare, but typically aggressive malignancy with an estimated annual incidence of 0.7–2.0 cases per million population. ACCs are highly aggressive with a poor prognosis and an overall survival rate at 5 years of only 25%–50% in most series [1, 2]. Until recently, radical surgical resection has been the only potentially curative option for ACC; however, approximately 40% of patients initially present with distant metastases. Even after seemingly complete removal of the tumor, recurrences occur in approximately 60%–80% of ACC patients [3]. Unfortunately, there is no effective therapy for patients with advanced or recurrent ACC. The combination of a cytotoxic drug and mitotane is recommended as first-line therapy in advanced ACCs, but the poor therapeutic efficiency (< 40%) and severe side effects limits clinical utility [4]. Therefore, new agents for ACC treatment are urgently required.

Rottlerin(1-[6-[(3-acetyl-2,4,6-trihydroxy-5-methylphenyl)methyl]-5,7-dihydroxy-2,2dimethyl-2H-1-benzopyran-8yl]3-phenyl-2-Propen-1-one), also known as mallotoxin, is a natural plant polyphenol. Rottlerin is a traditional Indian medicine previously used as an antagonist of fertilization and treatment for cestode and trematode infections [5]. In recent years, rottlerin has been shown to regulate several cell processes, including cell circle regulation, cellular proliferation, apoptosis, autophagy, and migration [6]. Numerous studies have demonstrated that rottlerin plays an important role in anti-tumor activity in several cancers like prostate cancers and pancreatic cancer, and has great potential as a novel chemotherapeutic agent. Other studies have shown that rottlerin suppresses tumor cell survival in breast cancer, likely via suppression of the Wnt/β-catenin signal pathway [7]. Within the adrenal cortex, the Wnt/β-catenin cascade is involved in adrenal development and differentiation [8]. Indeed, abnormal functioning of the Wnt/β-catenin signal pathway may lead to adrenal tumourigenesis [9, 10]. In patients with ACC, β-catenin activation is associated with decreased overall survival. The Wnt/β-catenin pathway is expected to be an efficient targeting strategy for the treatment of ACC [11, 12]. Therefore, we sought to investigate the potential role and preliminary mechanisms of rottlerin in tumor cell proliferation, migration, and invasion in ACC cell lines and an ACC xenograft mouse model. In addition, we explored the potential therapeutic utility of rottlerin as a novel and potential chemotherapeutic agent for ACC.

## RESULTS

### Rottlerin inhibits ACC cell proliferation *in vitro*

**Figure 1:**
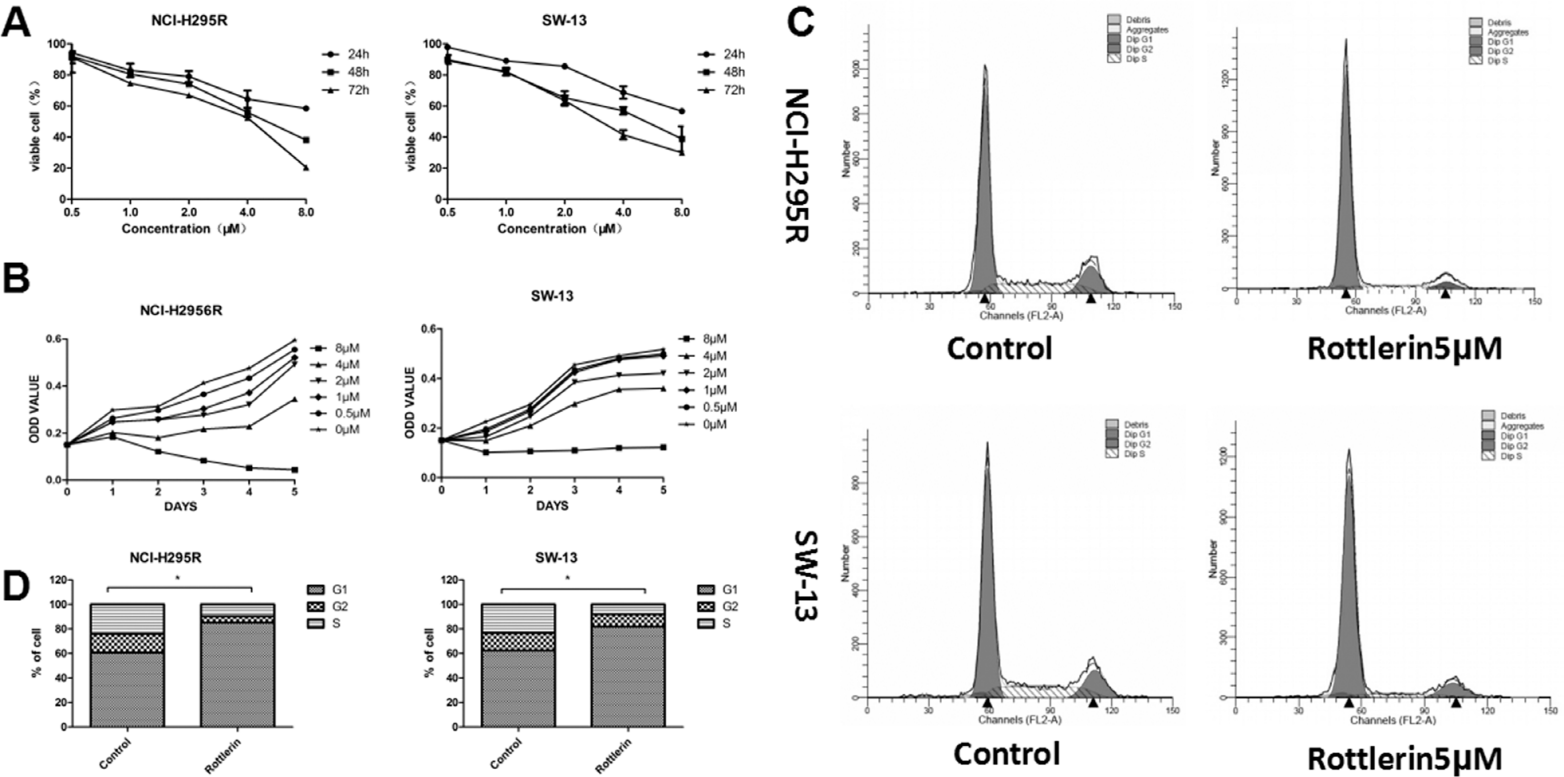
(**A**) Rottlerin both inhibited proliferation of ACC cells in a dose- and time-dependent manner. (**B**) Growth curve of NCI-H295R and SW-13 cells treated with rottlerin (0, 0.5, 1, 2, 4, and 8 μM) for 5 days. The growth of rottlerin–treated cells was relatively slower than untreated cells in a dose-dependent manner. (**C**) Rottlerin caused cell cycle arrest of ACC cells at the G0/G1 phase after treatment with 5 μM rottlerin for 48 h based on flow cytometric analysis. (**D**) Rottlerin caused cell cycle arrest of ACC cells at the G0/G1 phase compared with control (*p < 0.05).

To investigate the effects of rottlerin on ACC, we first evaluated whether or not rottlerin decreases viability in ACC cell lines. NCI-H295R and SW-13 cells were treated with rottlerin at the indicated concentration for 24, 48, 72, 96, and 120 h. It was shown that rottlerin inhibited proliferation of ACC cells in a time- and dose-dependent manner (Figure 1A). At a concentration of 4 μM, rottlerin inhibited approximately 44.0% and 43.2% of NCI-H295R and SW-13 cells, respectively, after treatment for 48 h. In contrast, when exposed to 8μM rottlerin for 72 h, the inhibition ratio (1-viable cell%) was up to 70% in SW-13 cells and 80% in NCI-H295R cells. The 5-day growth curves showed that the growth of rottlerin-treated cells was relatively slower than cells not treated with rottlerin in a dose-dependent manner (Figure 1B). The 48-h IC50 was 5.02 μM in NCI-H295R cells and 4.87 μM in SW-13 cells.

### Rottlerin induced ACC cell apoptosis *in vitro*

**Figure 2:**
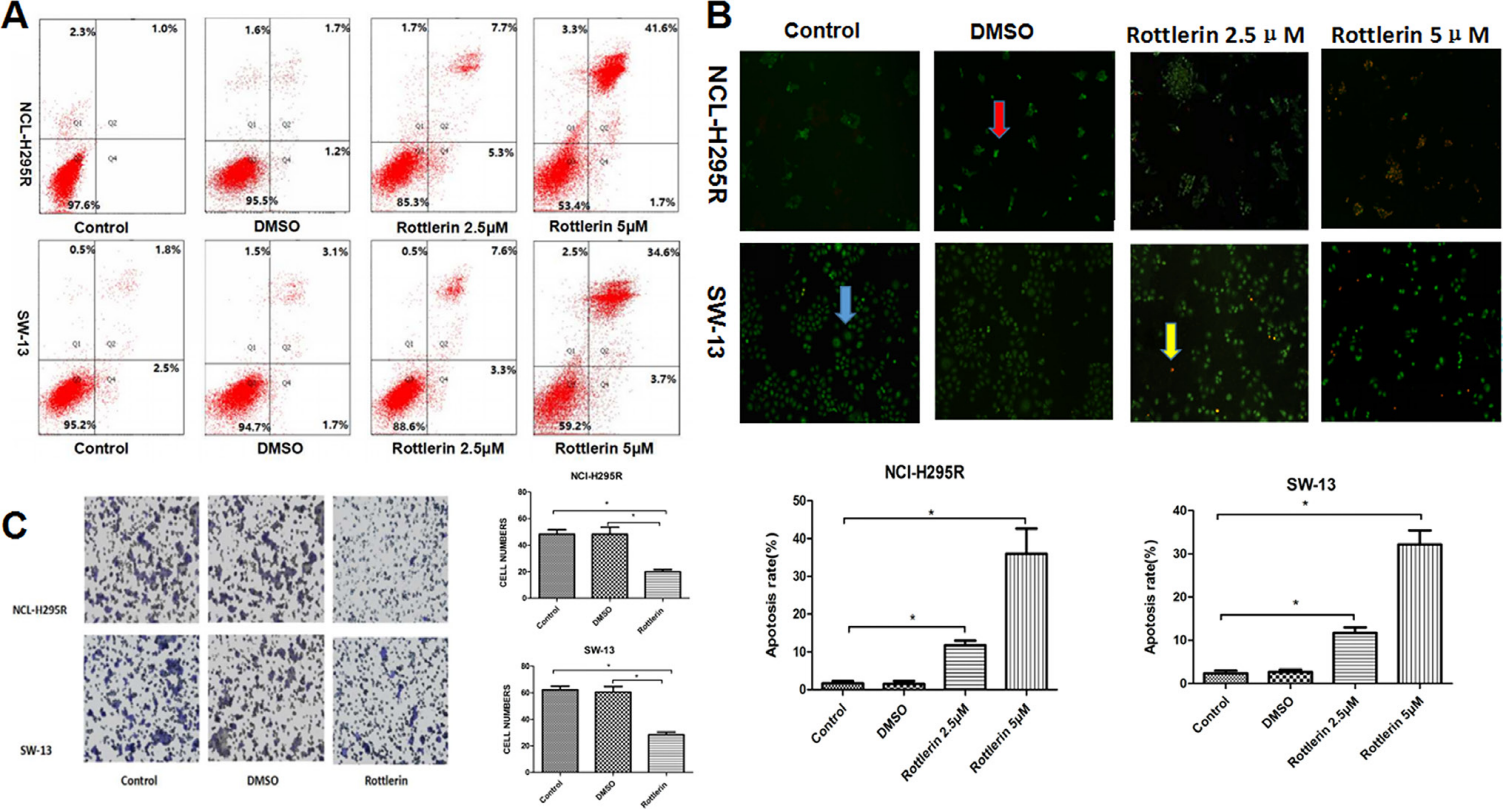
(**A**) Flow cytometric analysis of annexin-V/PI staining of NCI-H295R and SW-13 cells. Cells were treated with rottlerin at the indicated concentration (2.5 μM or 5 μM) for 48 h. The dual parameter dot plots combining annexin-V-fluorescein isothiocyanate (FITC) and PI fluorescence show the viable cell population in the lower left quadrant (annexin-V2, PI2), apoptotic cells in the lower right quadrant (annexin-V+, PI2) and upper right quadrant (annexin V+, PI+), and necrotic cells in the upper left quadrant (annexin-V2, PI+). (**B**) Morphologic changes of ACC cells based on AO/EB staining. After treatment with rottlerin (2.5 μM or 5 μM) for 48 h, apoptosis was observed using a fluorescence microscope. In order to indicate, the blue arrow point to a normal cell, the red arrow point to an early apoptotic cell, and the yellow arrow point to a late apoptotic cell. The rate of apoptosis in the rotterlin-treated group was higher compared with the control and DMSO groups (*p < 0.05). The apoptotic rate of cells in the higher concentration groups after treatment with 5 μM rottlerin for 48h was higher compared with the lower concentration groups (*p < 0.05). (**C**) The transwell invasion and migration assay showed that rottlerin inhibited cell invasion and migration of ACC cells (*p < 0.05).

To further elucidate the mechanism by which rottlerin inhibits cellular growth in ACC cells, we determined whether or not rottlerin induces apoptosis in ACC cells. Flow cytometry was performed to show the apoptosis rate of ACC cells after treatment with rottlerin at the indicated concentration (5 μM [approximately the 48 h IC50] and 2.5 μM [approximately half of the 48 h IC50]). The percentage of apoptosis (early apoptosis plus late apoptosis) was identified in NCI-H295R and SW-13 cells after rottlerin treatment (Figure 2A). The results showed an increase in the percentage of apoptotic cells in the rottlerin-treated group compared with the control and DMSO groups. After treatment with 2.5 μM rottlerin for 48 h, the percentage of apoptotic cells reached 13.0% in the NCI-H295R cells and 10.9% in the SW-13 cells. After treatment with 5 μM rottlerin for 48 h, the percentage of apoptotic cells reached 43.3% in the NCI-H295R cells and 38.3% in the SW-13 cells. The results suggest that rottlerin induces apoptosis in a dose-dependent manner. The results of AO/EB staining support these conclusions regarding morphology changes. The apoptosis rate increased in the rottlerin-treated group compared with the control and DMSO groups with AO/EB staining (Figure 2B, *p < 0.05). The apoptosis rate in the 5μM rottlerin-treated group was higher compared to the 2.5μM rottlerin-treated group (Figure 2B, *p < 0.05).

### Rottlerin caused cell cycle arrest of ACC cells *in vitro*

Rottlerin inhibited cellular proliferation and may also be involved with cell cycle arrest. Therefore, we investigated the effect of rottlerin on the cell cycle of ACC cells using flow cytometry analyses. After treatment with 5 μM rottlerin for 48 h, G0/G1 cell cycle arrest occurred in ACC cells (Figure 1C). The percentage of cells in the G1 phase was 85.14% in NCI-H295R cells and 81.88% in SW-13 cells, which was significantly increased compared with the control group (Figure 1D, *p < 0.05).

### Rottlerin inhibited cell invasion and migration of ACC cells

Invasion and migration are related to the malignant features of ACCs [13]. To determine if rottlerin inhibits cell invasion and migration of ACC cells, we performed transwell invasion and migration assays. In the rottlerin treatment group, the invasion of ACC cells was impaired by rottlerin (5 μM); significantly fewer cells migrated to the lower side of the transwell chamber membrane than the control group (Figure 2C, *p < 0.05).

**Figure 3:**
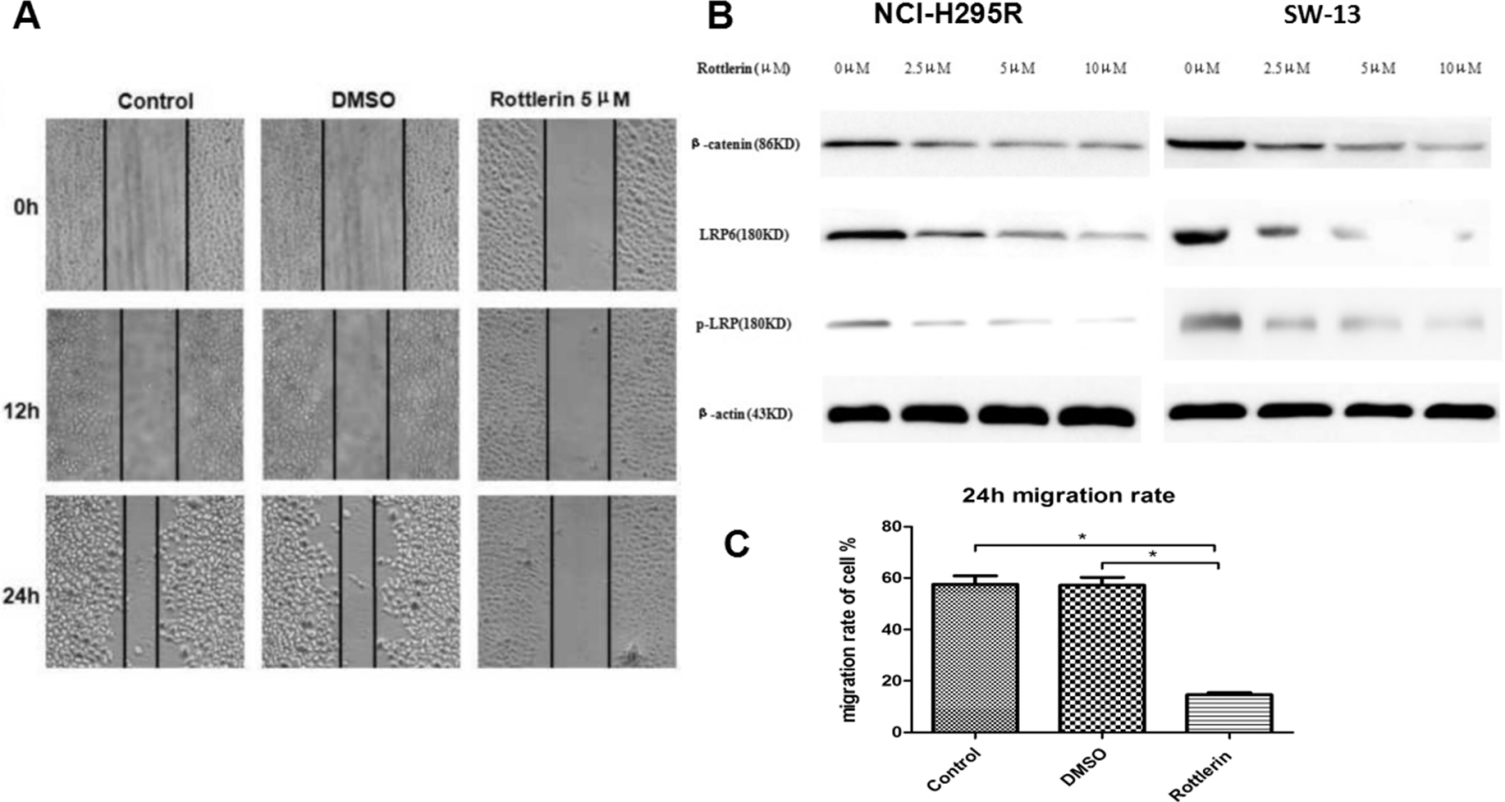
(**A**) The wound healing assay showed that rottlerin reduced the cell migration rate of SW-13 cells compared with the control and DMSO groups. (**B**) WB assays of β-catenin, LRP6, and p-LRP were performed in NCI-H295R and SW-13 cells. The results showed that rottlerin down-regulated the expression of β-catenin, LRP6, and p-LRP in a dose-dependent manner. (**C**) The 24 h migration of SW-13 decreased after rottlerin treatment compared with the control and DMSO-treated groups (*p < 0.05).

The results of wound healing assay demonstrated that the migration rate of SW-13 cells was significantly reduced by rottlerin (5 μM) compared with the control group (Figure 3A). The 24 h migration rate was significantly decreased in SW-13 cells (Figure 3C, *p < 0.05).

### Rottlerin inhibited tumor growth *in vivo*

**Figure 4:**
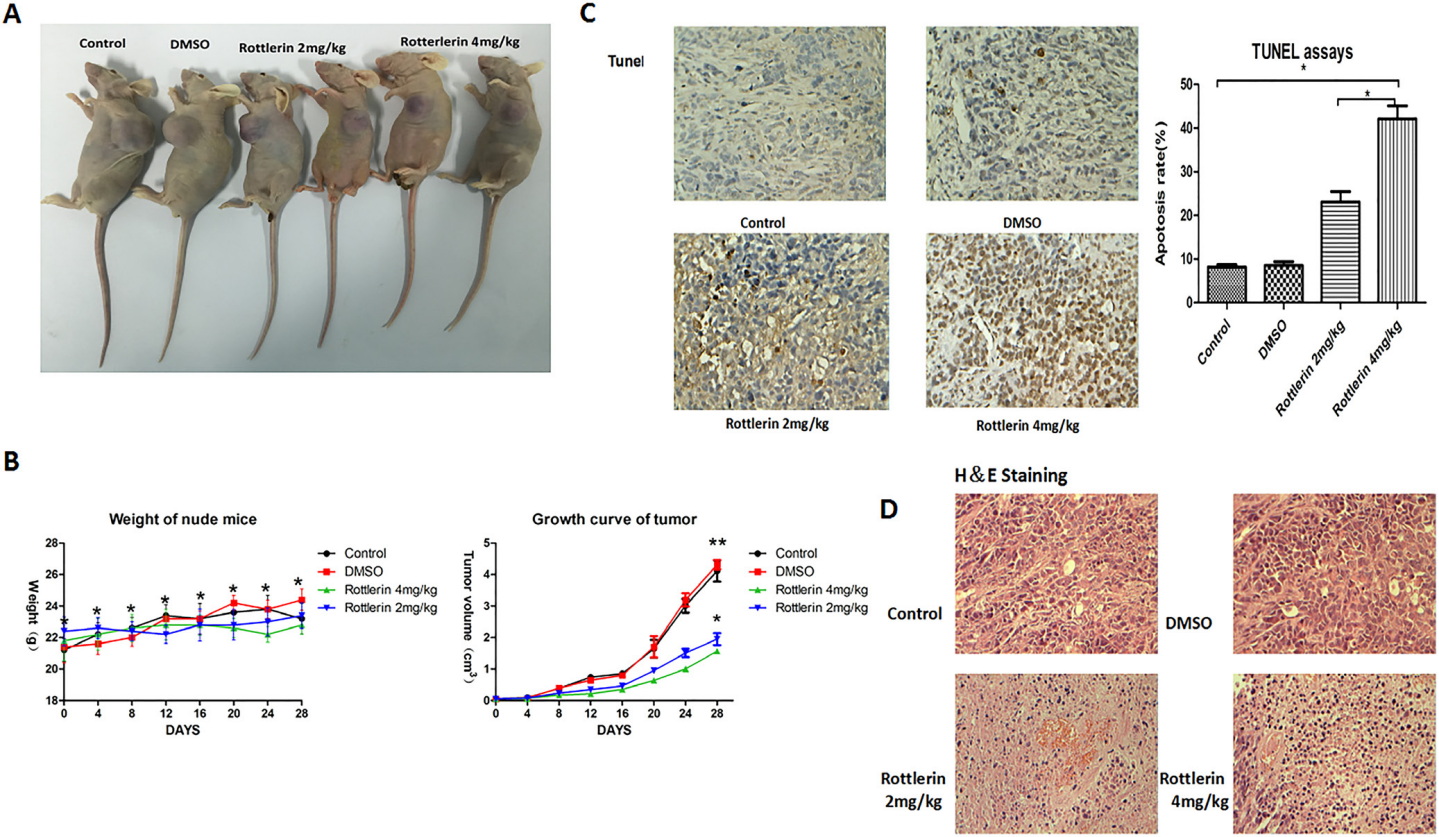
(**A**) Tumors in the rottlerin-treated groups were smaller than the control and DMSO-treated groups. (**B**) There was no significant difference in weight among the groups (*p > 0.05), and the rottlerin-treated groups showed delayed tumor growth compared to the control and DSMO-treated groups (*p < 0.05); there was no significant difference in tumor volume between the control and DSMO-treated groups (**p > 0.05). Tumors in the rottlerin 4 mg/kg group grow more slowly compared with the rottlerin 2 mg/kg group (*p < 0.05). (**C**) Tunnel assays indicated that rottlerin induced apoptosis *in vivo*. The tumor sections were observed under a light microscope. The apoptotic cells appeared brown in color, when the normal cells appeared blue. The apoptotic rates of the groups showed that the rottlerin-treated groups had higher apoptotic rates than the control and DMSO-treated groups (*p < 0.05). The apoptotic rate in the rottlerin 4mg/kg group was higher than the rottlerin 2 mg/kg group (*p < 0.05). (**D**) Hematoxylin and eosin (H&E) staining of tumor tissue.

**Figure 5:**
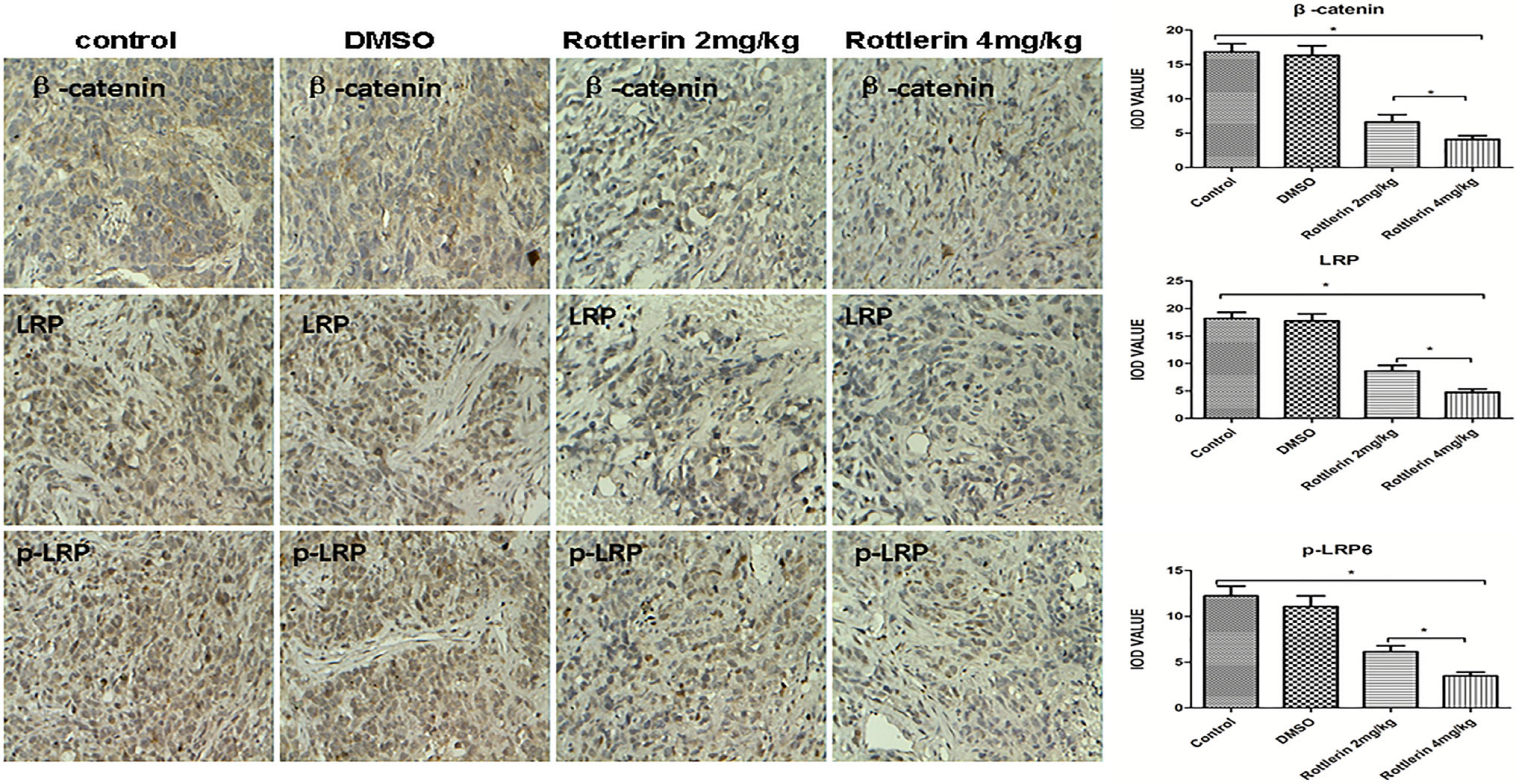
The expression of β-catenin, LRP6, and p-LRP by immunohistochemistry staining in xenograft tissues The positive expressing proteins appeared brown in color. The results showed that rottlerin down-regulated the expression of β-catenin, LRP6, and p-LRP compared with the control and DMSO-treated groups (*p < 0.0*5*) *in vivo*. The suppression was stronger in the higher dose-treated group (rottlerin 4 mg/kg) than the lower dose-treated group (rottlerin 2 mg/kg; **p < 0.05).

To investigate the effect of rottlerin on anti-tumor activities *in vivo*, we established a SW-13 xenograft tumor model in nude mice. With the administration of rottlerin for 4 weeks, tumor sizes and body weight were measured every 4 days. Assessment of tumor volume showed that the rottlerin-treated groups had delayed tumor growth compared to the control group and the group treated with DSMO (Figure 4A). There was no significant weight differences among the groups and there was no toxicity or side effects in the mice after 4 weeks of treatment (Figure 4B). Then, mice were sacrificed and the xenografts were extirpated. As shown in Figure 5, tumors were smaller in the experimental groups than the control and DSMO groups, and tumors were smaller in the rottlerin 4 mg/kg group than the rottlerin 2 mg/kg group. The mean volume of the tumors decreased from 3.23 cm^3^ (control) and 3.35 cm^3^ (DMSO) to 1.56 cm^3^ (rottlerin 4 mg/kg) and 1.94 cm^3^ (rottlerin 2 mg/kg); the difference was statistically significant (*p* < 0.05). TUNEL assays were performed to assess the anti-tumor activity of rottlerin *in vivo* (Figure 4C). The TUNEL assays indicated that the rottlerin-treated groups had higher apoptotic rates compared with the control and DMSO-treated groups.

### Rottlerin suppressed the Wnt/β-catenin signaling pathway

To determine if rottlerin suppresses the Wnt/β-catenin signaling pathway in ACC, and to elucidate the mechanism by which rottlerin mediates ACC cell apoptosis, some signaling molecules in the Wnt/β-catenin pathway were examined. Based on result of Western blotting, We can conclude that rottlerin reduced the expression of β-catenin, LRP6, and p-LRP in ACC cells in a dose-dependent manner (Figure 3B).

Furthermore, we performed H&E (Figure 4D) and immunohistochemistry staining in xenograft tissues, and the results showed that rottlerin also down-regulated the expression of β-catenin, LRP6, and p-LRP compared with the control and DMSO-treated groups *in vivo* (Figure 5, *p < 0.05). Moreover, in the higher dose-treated group (rottlerin 4 mg/kg), the expression of β-catenin, LRP6, and p-LRP was lower than the lower dose-treated group (rottlerin 2 mg/kg).

## DISCUSSION

There is no effective treatment for patients with locally advanced and metastatic ACC. Development of effective treatment or novel agents for patients with locally advanced and metastatic ACC is significant [14]. Rottlerin, which is extracted from *Mallotus philippinensis*, is a polyphenol that has been used as an ant-helminthic or a fertilization antagonist. Rottlerin has been used in humans for many years, has a good safety profile, and exhibits little toxicity, even after long-term exposure [5]. Recently, rottlerin has been shown to exhibit anti-neoplastic activity in several cancers, including breast, prostate, colorectal, and pancreatic cancers [7, 15, 16] by inhibiting various cellular pathways known to play important roles in carcinogenesis, like Wnt/β-catenin signaling pathway and mammalian target of rapamycin complex 1 (mTORC1) signaling.however, its antineoplastic activity in ACC has rarely been studied.

Wnt/β-catenin signaling plays an important role in embryonic development and can lead to tumor formation when aberrantly activated [14, 17]. The Wnt pathway has been examined in relation to many cancers, such as leukemia, melanoma, lung cancer, and colorectal cancer [18]. Activation of the Wnt/β-catenin pathway occurs in ACC and is frequently caused by activating mutations of the gene encoding Wnt/β-catenin [18]. Therefore, blocking the Wnt/β-catenin pathway is expected to be an efficient targeting strategy for the treatment of ACCs [4]. Recent studies have reported that rottlerin blocks the Wnt/β-catenin signaling pathway in some cancers, including colorectal, prostate, and breast cancers [7].

In this study, we examined the effect of rottlerin on ACC cells. The results showed that rottlerin inhibited proliferation, induced apoptosis and G0/G1 cell cycle arrest, and decreased invasion and mobility of ACC cell lines. Furthermore, the results of animal experiments showed that rottlerin inhibited tumor growth *in vivo*, and there was no obvious toxic effect in nude mice. The results showed that rottlerin has anti-neoplastic activity in ACC, and could serve as a novel agent for the treatment of ACC.

We then turned to study the mechanism underlying the antineoplastic activity of rotterlin against ACC. We detected the expression of several key components of the Wnt/β-catenin signaling pathway in ACC cell lines and xenografts of nude mice treated with rottlerin. Based on Western blot and immunohistochemistry staining, the expression of β-catenin, LRP6, and p-LRP6 was down-regulated after treatment with rottlerin. We deduced that rottlerin plays a role as an anti-tumor agent in ACC related to Wnt/β-catenin signaling pathway, likely via suppression of the Wnt/β-catenin signaling pathway.

β-catenin, which is encoded by the CTNNB1 gene, is a key component of this signaling pathway and has multiple functions, including mediation of cell adhesion and signal transduction [19, 20]. β-catenin combines with a variety of proteins to regulate cell proliferation and differentiation, which is critical for embryonic development and tumorigenesis [21]. In ACC, accumulation of β-catenin has frequently been noted, indicating activation of the Wnt signaling pathway. Mutations in β-catenin have been associated with a poor prognosis in patients with ACC, and higher-grade ACC is associated with higher β-catenin expression [22]. In our study,β-catenin was down-regulated by rottlerin in ACC cells, both *in vitro* and *in vivo*.

LRP6, an indispensable co-receptor for the Wnt signaling pathway, is a member of the low-density lipoprotein receptor family, which interacts with the seven transmembrane receptor of the Frizzled (Fzd) family to activate the Wnt/β-catenin signaling pathway [23]. LRP6 is expressed in human cancer cell lines and up-regulated in human malignant tissues [24], and LRP6 silencing weakens Wnt/β-catenin signaling and inhibits cell proliferation and tumor growth in breast and prostate cancers [25]. LRP6 is readily expressed in ACC cells [26], but there has been limited research involving LRP6 in ACC. In our study, LRP6 and its phosphorylated form (p-LRP6) were down-regulated by rottlerin in ACC cells, both *in vitro* and *in vivo*.

Blocking the Wnt signaling pathway is not the only mechanism underlying rottlerin anti-tumor activity. Some studies have indicated that rottlerin decrease carcinogenesis by impacting the mammalian target of rapamycin complex 1 (mTORC1) signaling [27]. Furthermore, rottlerin inhibits the markers of angiogenesis (COX-2, VEGF, VEGFR, and IL-8), and metastasis (MMP-2 and MMP-9), thus blocking production of tumorigenic mediators in the tumor microenvironment [28]. Rottlerin also inhibits epithelial-mesenchymal transition by up-regulating E-cadherin [29, 30]. According to these studies, we know that the anti-neoplastic activity of rottlerin is a multiple-signal pathway. The anti-tumor activity of rotterlin against ACC warrants further study.

The recommended chemotherapy regimens for ACC are etoposide +doxorubicin+cisplatin+mitotane (EDP/M) or streptozotocin+mitotane (Sz/M). Whatever the chemotherapy regimen, mitotane is a key agent; however, the effective rate of treatment is only approximately 40%. Moreover, mitotane has intense side effects, thus limiting its clinical use [31, 32]. In this study, we attempted to identify a novel agent which suppresses ACC more efficiently and safer than traditional therapy. Based on our results, rottlerin is a novel and potential chemotherapeutic agent for ACC based on multiple signal pathway-related anti-neoplastic activity.

Further studies should involve clinical trials better defining the anti-neoplastic activity of combination rottlerin and traditional chemotherapeutics or cytotoxic drugs.

## MATERIALS AND METHODS

### Cell lines and culture

The ACC cell lines, NCI-H295R and SW-13, were purchased from the Cell Repository of the Chinese Academy of Science and originated from the American Type Culture Collection (ATCC; Manassas, VA, USA). The NCI-H295R cell line was cultured in DMEM (GIBCO, Grand Island, NY,USA), supplemented with 10% fetal bovine serum (MPBIO, Carlsbad, CA, USA) in a standard humidified incubator at 37°C in a 5% CO_2_ atmosphere. The SW-13 cell line was cultured in L15 media (GENOM, Shanghai, China) supplemented with 10% fetal bovine serum in a standard humidified incubator at 37°C in an atmosphere without CO_2_.

### Drugs and antibodies

R00ottlerin was purchased from Sigma Aldrich (St. Louis, MO, USA). Rottlerin was dissolved in dimethyl sulfoxide (DMSO; GIBCO) at 10mM and stored in dark at 4°C. The final concentrations of rottlerin used for different experiments were prepared by diluting the stock solution with media solution. The antibodies used for Western blotting and immunohistochemistry staining were as follows: anti-LRP6 and anti-p-LRP6 antibodies were purchased from Affinity Bioscience (Cincinnati, OH, USA); and anti-β-catenin antibody was purchased from ABCAM (San Francisco, CA, USA).

### Cell viability

NCI-H295R and SW-13 cells were plated at a density of 2000 cells/well in flat bottom 96-well plates (100μl media per well). After 24 h, cells were treated with rottlerin at various concentrations (0, 0.5, 1, 2, 4, and 8 μM) in the dark. Cell viability was measured after 24, 48, 72, 96, and 120 h using the Cell Counting Kit 8 (Dojindo, Osaka, Japan) with a microplate reader at 450 nm. All experimental concentrations were assessed in triplicate. The half maximal inhibitory concentration (IC50) was calculated with GraphPad Prism 5.

### Apoptosis analyses by flow cytometry

NCI-H295R and SW-13 cells were cultured in 6-well plates and treated with rottlerin in the dark (concentration : 5 μM and 2.5 μM ). The control group was not treated with rottlerin and a DMSO treated-group was established as well. After incubation for 48 h, cells were harvested, washed with PBS twice, resuspended in binding buffer, stained with an annexin-V/PI solution (Dojindo) at room temperature, then analyzed using a FACScan system (BD Biosciences, San Jose, CA, USA).

### Acridine orange/ethidium bromide (AO/EB) staining

Morphologic assessment of apoptotic cells was performed using the AO/EB staining method. NCI-H295R and SW-13 cells were cultured in 6-well plates (5 × 10^4^ cells/ml). After a 24-h incubation, the cells were treated with rottlerin in the dark (concentration = 5 μM and 2.5 μM) for 48 h. After washing twice with PBS, the cells were stained with 1ml of AO/EB (Sigma Aldrich) for 3min and imaged under an inverted fluorescence microscope. Five hundred cells were counted under a microscope (magnification = 200×). The apoptosis rate was calculated using the following formula: apoptosis rate = (early apoptotic cells + late apoptotic cells)/the total number of cells. The nuclei of early apoptotic cells had a bright green fluorescence, the late apoptotic cell nuclei had a bright orange fluorescence, and the nuclei of normal cells had a ground glass-like dim green fluorescence.

### Cell cycle analyses by flow cytometry

NCI-H295R and SW-13 cells were plated in 6-well plates. After a 24 h incubation, the cells were treated with rottlerin in the dark (concentration = 5 μM and 2.5 μM) for 48 h. The cells were collected, washed twice in cold PBS, mixed in 300 ml of 1×binding buffer, and incubated at room temperature for 15 min with propidium iodide (PI), NP-40, and RNaseA (Beyotime, Shanghai, China). The cell cycle was analyzed by flow cytometry and the percentage of cells in the different phases was calculated using ModFit LT software (Verity Software House).

### Invasion and migration assay

Cell invasion and migration were assessed using a Transwell chamber (GreinerBio, Frickenhausen, Germany) containing a polycarbonated filter with 8lM pores coated with Matrigel (BD Biosciences, BD Biosciences, San Jose, CA, USA). The cells in the experimental group were pre-treated with the respective concentrations of rottlerin (5 μM) for 24 h. The cells (1 × 10^5^) in 0.2 ml of culture medium without fetal bovine serum were added to the upper chamber. The lower chamber was filled with 0.6 ml of complete medium containing 30% fetal bovine serum. After a 24 h incubation,cells remaining on the upper side of the transwell membrane were removed using a cotton swab. The membrane was washed with ice cold PBS twice. Cells that invaded the underside were fixed with 4% formaldehyde, stained with crystal violet, and counted in five randomly selected fields under a microscope (magnification of 200×).

We also measured the migration rate using a wound healing assay. SW-13 cells were plated in 6-well plates (1 × 10^6^cells/ml); after attachment the cells were scratched using a sterile pipette tip. The experimental group was treated with 5μM rottlerin. The cells were photographed at various time points (0, 12, and 24 h; magnification = 100×). The migration rate at 24 h was calculated using the following formula:migration rate = (1-the width of the cell wound at 24 h/the width of the cell wound at 0 h)%.

### Animal experiments

Animal experiments were performed using 4-week-old male nude mice (athymic, BALB/C nu/nu), which were purchased from the Provincial Animal Center (Guangdong, China). The animal experiments were performed according to the Guide for the Care and Use of Laboratory Animals and approved by the Animal Investigation Committee of the General Hospital of Guangzhou Military Command. Mice were housed in a standard animal laboratory with free access to water and food. The mice were kept under constant environmental conditions with a 12 h light-dark cycle. SW-13 cells were (3 × 10^6^ cells) suspended in 200 μl of serum-free culture medium and inoculated subcutaneously in the flank region of nude mice. Tumors were permitted to grow to approximately 4mm, and mice were randomized into 4 treatment groups (control, DMSO, rottlerin 4 mg/kg, and rottlerin 2 mg/kg) with 5 mice per group. All the mice were injected intraperitoneally daily for 4 weeks. Rottlerin was dissolved in DMSO, and diluted with normal saline (NS). The control group was injected with 0.5 ml of NS and the DMSO group was injected with 0.45 ml of NS mixed with 50 μl of DSMO. The rottlerin groups were injected with rottlerin (4 mg or 2 mg per kilogram). The mouse weights and tumor volumes were monitored every 4 days. Using Vernier calipers, the tumor volume was calculated according to the following formula: A × B × B/2, where A is the length of the tumor and B is the width. After 4 weeks, the mice were euthanized and the xenografts were removed from the animals. Tumor tissues were fixed in 10% formalin solution and paraffin-embedded. The sample sections were prepared for hematoxylin and eosin (HE) staining, immunohistochemistry staining, and terminal deoxynucleotidyl transferase dUTP nick end labeling (TUNEL) assays.

### Immunohistochemistry staining

Tumor tissues sections from paraffin-embedded tumors were de-paraffinized and rehydrated using xylene and ethanol, and immersed in 3% hydrogen peroxide solution for 10 min to block endogenous peroxidase. Sections were boiled for 30 min in 10 mM citrate buffer solution (pH 6.0) for antigen retrieval. Slides were incubated for 45 min with 5% bovine serum albumin and incubated overnight at 4°C with anti-LRP6, anti-p-LRP6, and anti-β-catenin antibodies. The specimens were incubated for 45 min at 37°C with the appropriate peroxidase-conjugated secondary antibody and visualized using the DAB Detection Kit (BOSTER, Shanghai, China) following the manufacturer's instructions. All sections with immunohistochemical staining were observed and the pictures of 6 randomly selected but homogeneous in staining and cell numbers fields (400×) were photographed by an Olympus microscope (IX-70 OLYMPUS, Japan) under high power view. The integrated optical density (IOD) in each image was measured with the same setting for all the slides, and the results were analyzed with Image-ProPlus 6.0 software[33].

### TUNEL assays

Tumor tissue sections from paraffin-embedded tumors were de-paraffinized and rehydrated using xylene and ethanol. The slides were rinsed twice with PBS and treated for 15 min at 37°C with proteinase K (15 mg/ml in 10 mM Tris/HCl [pH 7.4–8.0]). Endogenous peroxidases were blocked using 3% hydrogen peroxide in methanol at room temperature for 10 min. The tissue sections were then analyzed with an *in situ* Cell Death Detection Kit-POD (Roche, Basel, Switzerland) in accordance with the manufacturer's instructions. The reaction was visualized using microscopy. Cell numbers of five randomly selected fields(400×) were counted under a microscope and analyzed with Imaga-ProPlus 6.0 software.

### Western blotting

NCI-H295R and SW-13 cells were treated with rottlerin in the dark (concentrations = 0, 2.5, 5, and 10 μM) for 48 h. The cells were washed twice with PBS solution, then lysed with RIPA Lysis Buffer (Beyotime, Shanghai, China) and a protease inhibitor (Thermo Scientific, Waltham, MA, USA). Protein concentrations were determined with a Pierce BCA Protein Assay Kit (Beyotime). Equivalent amounts of total protein (60 μg) were boiled and electrophoretically separated on a 10% polyacrylamide gel at 80 volts. The proteins were transferred to a polyvinylidene difluoride membrane. The membranes were blocked for 60 min with a 5% milk solution prepared in PBS. The membranes were incubated overnight at 4°C with 1:500 (anti-p-LRP6 and anti-LRP6) or 1:3000 (anti-β-catenin) dilutions of the primary antibodies. The membranes were washed three times for 5 min each with Tween 20 (1:1000 dilution in PBS) and incubated for 45 min with the appropriate peroxidase-conjugated secondary antibody (1:5000 dilution). The membranes were washed with Tween 20-PBS three times for 10 min each and were developed using an Odyssey two-color infrared laser imaging system. The signal generated by β-actin was used as an internal control.

### Statistics

Statistical analyses were performed using SPSS17.0 software and all the data from at least three experiments are presented as the mean ± S.D. The statistical difference between the means was analyzed with a Student's *t-test* or one-way ANOVA. Compared with the control, a *p <* 0.05 was considered to be statistically significant.
